# Mapping progress in intravascular catheter quality surveillance: An Australian case study of electronic medical record data linkage

**DOI:** 10.3389/fmed.2022.962130

**Published:** 2022-08-11

**Authors:** Jessica A. Schults, Daner L. Ball, Clair Sullivan, Nick Rossow, Gillian Ray-Barruel, Rachel M. Walker, Bela Stantic, Claire M. Rickard

**Affiliations:** ^1^Alliance for Vascular Access Teaching and Research Group, Nathan, QLD, Australia; ^2^School of Nursing, Midwifery and Social Work, The University of Queensland, St Lucia, QLD, Australia; ^3^Metro North Health, Herston Infectious Disease Institute, Herston, QLD, Australia; ^4^School of Information and Communication Technology, Griffith University, Nathan, QLD, Australia; ^5^Digital Metro North, Metro North Hospital and Health Service, Herston, QLD, Australia; ^6^Centre for Health Services Research, Faculty of Medicine, University of Queensland, Herston, QLD, Australia; ^7^Digital Solutions, Griffith University, Nathan, QLD, Australia; ^8^School of Nursing and Midwifery, Menzies Health Institute Queensland, Griffith University, Nathan, QLD, Australia; ^9^Division of Surgery, Princess Alexandra Hospital, Brisbane, QLD, Australia; ^10^Nursing and Midwifery Research Centre, Royal Brisbane and Women’s Hospital, Brisbane, QLD, Australia

**Keywords:** intravascular catheter, quality surveillance, electronic health record, clinical informatics, patient – centered care

## Abstract

**Background and significance:**

Intravascular (IV) catheters are the most invasive medical device in healthcare. Localized priority-setting related to IV catheter quality surveillance is a key objective of recent healthcare reform in Australia. We sought to determine the plausibility of using electronic health record (EHR) data for catheter surveillance by mapping currently available data across state-wide platforms. This work has identified barriers and facilitators to a state-wide EHR surveillance initiative.

**Materials and methods:**

Data variables were generated and mapped from routinely used EHR sources across Queensland, Australia through a systematic search of gray literature and expert consultation with clinical information specialists. EHR systems were eligible for inclusion if they collected data related to IV catheter insertion, care, or outcomes of hospitalized patients. Generated variables were mapped against international recommendations for IV catheter surveillance, with data linkage and data export capacity narratively summarized.

**Results:**

We identified five EHR systems, namely, iEMR, MetaVision ICU^®^, Multiprac, RiskMan, and the Nephrology Registry. Systems were used across jurisdictions and hospital wards. Data linkage was not evident across systems. Extraction processes for catheter data were not standardized, lacking clear and reliable extraction techniques. In combination, EHR systems collected 43/50 international variables recommended for catheter surveillance, however, individual systems collected a median of 24/50 (IQR 22, 30) variables. We did not identify integrated clinical analytic systems (incorporating machine learning) to support clinical decision making or for risk stratification (e.g., catheter-related infection).

**Conclusion:**

Current data linkage across EHR systems limits the development of an IV catheter quality surveillance system to provide timely data related to catheter complications and harm. To facilitate reliable and timely surveillance of catheter outcomes using clinical informatics, substantial work is needed to overcome existing barriers and transform health surveillance.

## Introduction

Within the last decade, complications associated with intravascular (IV) catheters have increased in focus as new evidence reveals the burden of catheter failure in hospitalized patients (1, 2). Globally, IV catheters are the most prevalent invasive medical device used in healthcare (3, 4). Around 200 million catheters are used in the United States annually (5, 6) and approximately one in three United Kingdom inpatients have a peripheral IV catheter *in situ* (7, 8). Despite their widespread use, IV catheter-related complications are common and associated with increased clinical and economic burden, including increased mean hospitalization cost, protracted length of stay, and greater risk of death than patients without such complications (9–11).

Measurement of process, system, and outcome parameters related to IV catheters has been performed in hospitals using basic audit methods (12) with varied success for decades. This approach to quality surveillance is resource intensive and limits multijurisdictional benchmarking (13) and performance reporting (14) due to heterogeneity in items and case definitions (15). However, the reliability and validity of this data are becoming increasingly important with an increasing regulatory environment and persisting patient safety problems related to IV catheters. In 2021, the Australian Commission on Safety and Quality in Healthcare released a National Strategy to reduce the impact of IV catheter-related complications on hospitalized Australians. The clinical care standard sets out several quantifiable targets against which hospitals can measure quality and progress on IV catheter improvement initiatives. With the recent publication of international recommendations for vascular access surveillance (a minimum dataset; 16) there is an opportunity for jurisdictions to standardize IV catheter surveillance initiatives. This has led to health systems increasingly seeking platforms to routinely monitor patient and service-level outcomes and seek out new technologies to support enhanced healthcare quality and safety (17).

The broad adoption of electronic health records (EHRs) across the continuum of care has seen new opportunities arise to support health services monitor IV catheter quality. As a result, an increasing number of health professionals and researchers are now leveraging data sets from routine clinical care to improve health outcomes (18). However, the diversity and complexity of EHR data sets have created challenges, specifically in the collection and comparison of data items for effective analysis of quality and safety queries (19). Data linkage, interoperability, and heterogenous data items have been flagged as barriers to the implementation of standardized surveillance platforms in many health disciplines. However, these challenges have yet to be fully explored in IV catheter care provision (13), which crosses disciplines. An IV surveillance platform would depend largely on the fundamental structural design and utilization of relevant datasets (20, 21). Data captured on EHR platforms can yield powerful insights (17, 22, 23), such as evaluation of IV catheter quality improvement initiatives (24). Therefore, we sought to map, in an Australian case study, EHR data sources that collect indwelling IV catheter data to explore the benefits, challenges, and future directions of digitally enabled quality surveillance. Secondary aims include (1) comparing currently collected data variables against international recommendations for IV surveillance and monitoring; and (2) determining the current scope of clinical analytic frameworks integrated with EHRs related to IV catheters.

## Materials and methods

### Design

We conducted a scoping review and modified mapping study (25–27) to identify EHRs and IV data variables across Queensland Health systems. The main focus of these methods was classification by conducting thematic analysis. The search strategy included two components, a gray literature search and consultation with EMR experts, to answer the research questions (Supplementary material 1). The study was underpinned by the EHR Usability Evaluation and Use Case Framework by the Agency for Healthcare Research and Quality (28). Ethical approval was obtained from Griffith University (GU Ref: 2020/710).

### Setting

The study was conducted in Queensland, Australia. Located in northeastern Australia, the State of Queensland has an estimated population of 5.16 million (29). Public health services in Queensland are managed by Queensland Health and are funded by both the state and federal governments. At the time of study undertaking, integrated electronic medical record (ieMR) had been successfully implemented in 16 of 30 Queensland Health facilities, with regular application updates slowly digitalizing the previous paper-based processes (30). All 16 hospitals use the same systems based on the initial iEMR configuration rolled out and piloted in a single Queensland site.

### Search for gray literature

Our gray literature search comprised a (1) targeted search of known clinical datasets through government and healthcare agencies (national health agencies and safety and quality organizations), and (2) a Google search on the topic. For the latter, we adopted best practices for web-based searching in health research (31). EHRs were identified by key word searches including IV catheter, vascular access, central venous access device, peripherally inserted central catheter, healthcare-associated infection, central line associated bloodstream infection, and clinical/device/quality registry. The inclusion criteria were limited to studies that discussed EHRs. The broad search strategy was employed to help overcome anticipated barriers to locating data items including different data formats (different proprietary formats) and data linkage (variable scope of individual data sources and different data sources used within the health service; 31, 32). If further information was required to determine the eligibility of identified platform, the dataset manager was contacted *via* email correspondence. EHR eligibility criteria were: (1) currently utilized electronic or digital platform, and (2) minimum dataset included items related to indwelling IV catheter care, processes, or outcomes.

### Consultation with clinical information experts

Following ethics approval and literature search results, we consulted with 12 clinical information specialists (CISs; 20 contacted; 60% response rate), to augment and validate mapped EHRs. We received input from platform owners (e.g., Ocean Health Systems, HammondCare), health services (e.g., Metro North Hospital and Health Service CISs), Health and research institutes, and specialist providers (e.g., the Vascular Access Surveillance Team, Infection Control, and Intensive Care Unit) with deep content expertise in IV catheters. Purposive sampling with snowballing was used with potential experts identified through investigator networks. Experts were contacted *via* email (maximum 2 emails) or *via* phone with a short request to provide feedback on a working list of EHRs and IV catheter data elements contained in each system. We asked them to help identify any gaps in electronic data sources that might collect statewide IV catheter data. Participants were contacted in November 2020 and requested to respond within a month. If participants did not respond, it was assumed that they declined to participate in the study. Data sources identified through expert consultation were reviewed independently by two investigators (DB and JS) with discrepancies resolved by a third investigator (CS).

### Collating, summarizing, and reporting the results

All identified EHRs and data items were collated using a standardized data extraction form in Microsoft Excel^®^. We were interested in capturing broad features of EHRs including:

• EHR source host.
• Intravascular catheter data items and their definitions.
• Linkage capabilities.

The synthesis and classification scheme comprised the following steps: (1) enumeration of the number of EHRs and the number of items per EHR source; (2) presenting the visualization of the EHRs and data items included in the analysis; and (3) presenting a narrative summary of the principal findings (33). Finally collected IV catheter data items were mapped against international recommendations for IV catheter quality surveillance to determine alignment (see Supplementary material 2, republished with permission; 16).

## Results

### Mapping question 1. Which electronic health records collect intravascular catheter data across Queensland health?

**FIGURE 1:**
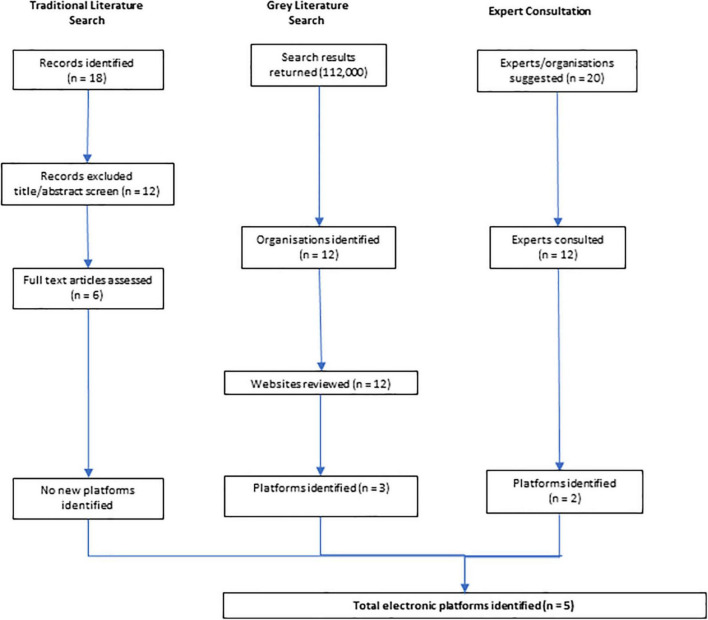
Flow chart of search results.

We identified 5 EHRs, namely, ieMR, MetaVision ICU, Multiprac, RiskMan, and the Nephrology Registry. As no ongoing linkages existed between datasets, the analyses presented represent EHR-level reports for each separate system (Figure 1). The gray literature search identified three EHRs; consultation with the experts identified a further two. Table 1 outlines the EHRs, host organizations, data source features, and availability of data.

**TABLE 1:** Electronic health record data sources.

Electronic data source; host	Data capture	Storage	Reporting
integrated electronic Medical Record (ieMR); Cerner^®^	Patient data is entered manually by clinicians during routine patient care.	Data is stored on Queensland Health corporate servers.	Vascular access specialists are able to export ieMR data into Microsoft Excel to track IV catheters within their local or health service context. *Data not publicly available.
MetaVision ICU; iMDSoft^®^	MetaVision ICU captures data from clinicians *via* manual data entry during routine care.	Data is stored on Queensland Health corporate servers within the public healthcare system and on private servers in the private healthcare sector.	Structured query language (SQL) automatically downloads patient data from MetaVision ICU for reporting and allows for specific reporting related to patient care including IV catheter data such as insertion and dwelling time. *Data not publicly available.
Multiprac; Ocean Health Systems	While some patient data are automatically downloaded, clinicians also input data manually into the program.	Multiprac data is stored internally within each hospital and health service. In the public sector (but not private), data is stored within Queensland Health.	Multiprac can produce reports for data collection for the organization, facility, specialty, unit and ward. The data collected is then reportable currently *via* crystal reports or as a downloaded xl/pdf extract. *Data available state-wide to infectious diseases specialists working in Queensland Health; not publicly available.
Nephrology; Queensland Health	Data is manually entered into the registry by clinicians as part of routine care.	Nephrology data is stored on a Microsoft Access database on servers housed within each hospital and health service.	Structured query language (SQL) automatically downloads patient data from Nephrology for reporting and allows for specific reporting related to patient care including IV catheter data such as insertion and dwelling time. *Data not publicly available.
RiskMan; Hammond Care	Reliant on clinicians manually enter adverse events data into the platform.	Data is stored on individual hospital and health service servers.	RiskMan is unable to report on IV catheter outcomes as it is reliant on the reporter to enter information relating to vascular access devices into the free text fields section of the platform. Based on communication with HammondCare and Queensland Health Quality and Safety staff, RiskMan is used and accessible to all health care providers across Queensland in public and private sectors. *Data not publicly available.

### Mapping question 2. What intravascular catheter data are currently collected across electronic health records, and how do they align with international recommendations?

**FIGURE 2:**
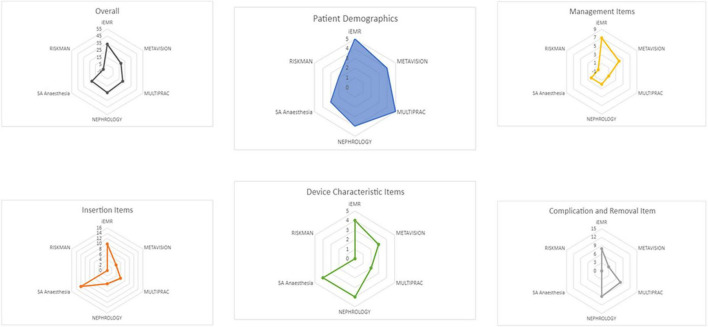
Intravascular catheter data items collected in Queensland electronic data sources compared to international recommendations.

The number of IV catheter items collected across EHRs was limited and focused on device characteristics (e.g., device type, insertion site; Figure 2). Cerner applications collected the most data items (PowerChart 38/50; 76%) in line with the 50-item international recommendations. EHRs collected a median of 24/50 (IQR 22, 30) variables. MetaVision ICU collected 30/50 (60%). The Nephrology Registry dataset collected 24/50 (48%) variables, while Multiprac collected 21/50 (42%) and RiskMan collected 2/50 (4%). Collectively, 43 of the 50 recommended items were captured. Items not collected included: (i) pain relief, (ii) blood sampling, (iii) line fracture, (iv) replacement required, (v) internal malposition, and (vi) can the patient identify the reason for the device?

Systematized Nomenclature of Medicine—Clinical Terms were embedded in ieMR and Multiprac, but not in MetaVision ICU, RiskMan, or Nephrology platforms. Few platforms had keyword search capacity for IV catheter, central venous access device, peripherally inserted central catheter, healthcare-associated infections, central line associated bloodstream infections, and/or clinical/device/quality registry. No EHR included mandatory fields (which a clinician must complete) related to IV catheter insertion, management, complications, or removal.

### Mapping question 3. What clinical decision support systems are currently in use to support intravascular catheter surveillance and care?

We identified no clinical decision support systems for IV catheters within the included electronic data platforms.

### Data sharing and linkage possibilities

All five EHRs were developed and are owned by separate enterprises (Cerner, iMDSoft, Ocean Healthcare Systems, HammondCare, Queensland Health). PowerChart, MetaVision ICU, RiskMan, and Multiprac systems currently link with the Hospital Based Corporate Information System (HBCIS), a statewide patient administration system that captures admitted and non-admitted patient, clinical, and administrative data. Multiprac additionally automatically draws positive bloodstream infection data from AUSLAB (statewide pathology system). The Nephrology Registry does not link to any systems within Queensland Health, as the platform was built on legacy database software that is no longer supported and is incompatible with the current version of Microsoft Windows software. As a result, clinicians must manually insert patient identifiers to track devices. At present, Cerner applications (ieMR) link with other Cerner applications within Queensland Health. MetaVision ICU (iMDSoft) links with HBCIS but not ieMR, meaning that ICU clinicians input replicated data into the Cerner platform, resulting in inefficiencies and duplication of clinical data for patients transitioning from ICU to the wards.

### Data extraction and reporting capability

**FIGURE 3:**
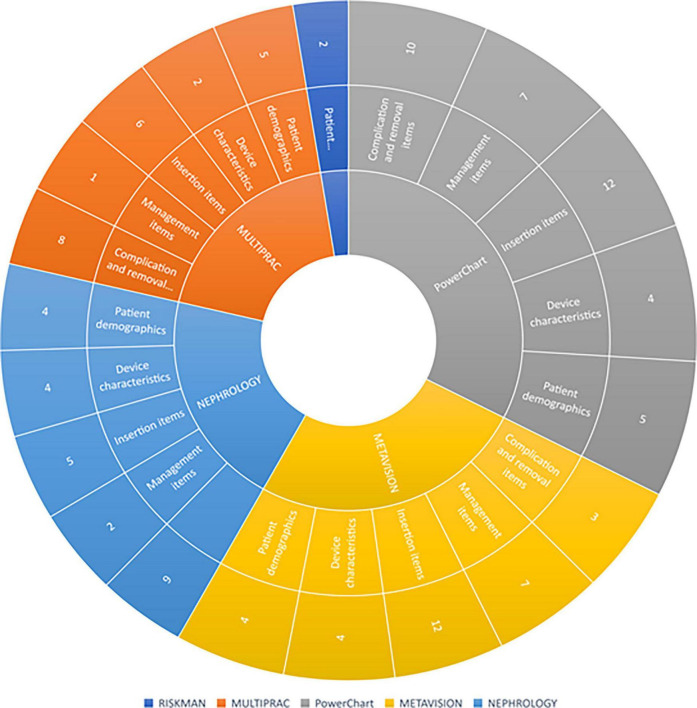
Sunburst diagram of vascular access items collected in Queensland Health electronic platforms. Each concentric circle represents a level of navigation from the top of the tree. In the majority of platforms, data items are displayed separately on different “pages” or “screens” from other items, requiring the user to view multiple screens to get a comprehensive clinical overview of vascular access items collected.

As per Figure 3, IV catheter data consistent with international recommendations can be extracted from all platforms, with the exception of RiskMan. The PowerChart (inpatient application) component of the ieMR automatically extracts IV catheter device and line data from SA Anesthesia into a procedure tab within the interactive view, however, it does not extract data into the lines and devices tab within Powerchart. Multiprac users tracking bloodstream infections can generate reports on positive bloodstream infection data for the organization, facility, specialty, unit, and ward. The data is then reportable currently *via* crystal reports, a Windows-based report writer solution allowing users to create specific reports, or as a downloaded Excel or portable digital file extract. Intensive care unit clinicians can track MetaVision ICU data through the structured query language server reporting service, which automatically extracts data from MetaVision ICU and generates a daily report for vascular access monitoring. Renal transplant clinicians can generate reports from the Nephrology Registry dataset through the SQL, which automatically extracts data for reporting. As RiskMan is a risk management and prevention database that relies on reporters entering data relating to vascular access devices into free text report fields, it is unable to generate reports.

## Discussion

In this scoping and mapping project of electronic surveillance capability for IV catheter usage, we identified five EHRs that capture IV catheter-related data across Queensland Health. Across all EHRs, 86% of internationally recommended quality surveillance items are collected, however, unlinked systems mean health jurisdictions are unable to utilize these systems to their maximum potential for catheter quality surveillance requirements. Importantly, patient reported experience measures were not captured across any EHR (e.g., can the patient identify the reason for the device; 14), a notable limitation of existing systems. Our findings confirm that despite the rapid digitalization of health care, many EHR systems are yet to reach their full potential to support hospital quality surveillance and proactive monitoring of IV catheter care. The effects of catheter quality improvement and harm prevention initiatives should be measured using data derived from reliable, standardized EHR surveillance programs. Such an EHR could support the development of clinical decision support tools for vascular access, and the testing of algorithms with the use of real-time clinical data (34).

A key finding of this study was that semantic interoperability (the ability of systems to exchange data with a shared meaning) will be a challenge for health systems in the future when integrating IV catheter data across EHRs (35). Different platforms and terminologies for expressing the same data entry (e.g., one type of catheter complication was variously described as puffy, leaking, infiltration, or extravasation) limit the aggregation and extraction capacity of patient data. Although reflective of the Queensland landscape, our findings are consistent with national and international reports, which highlight a “mismatch” in data terminology and interactions in the healthcare context (13, 36). This problem is not unique to IV catheters. It is likely that in time, programmers will adapt to existing platforms, addressing issues such as data fragmentation and ineffective interaction or display features to reflect clinical assumptions and workflows (37, 38). However, for now, this finding can be interpreted as a barrier to the exportation and use of clinical data to inform clinical decision support tools and improve the quality of vascular access care.

The potential of clinical analytics and support decision making in the context of IV catheter care is yet to be realized. We are yet to take advantage of emerging technologies (intelligent learning systems and trial-integrated clinical decision support software) to stratify patients’ risk of IV catheter complications (e.g., blood-stream infection). While this limitation is largely due to the feasibility of integrating this technology with current systems, progressing this advanced clinical technology would require improved data linkage and data exportation processes across EHR systems. An increasing number of researchers are developing predictive models for catheter complications (39–41), however, the practical application and value of machine learning models to reduce IV catheter complications and adverse health outcomes are yet to be demonstrated. Several international institutions (42, 43) have created IV catheter databases for a variety purposes such as risk factor analysis, health economic evaluations, and hospital-specific information (outcomes; 44, 45). Yet in Australia, HER surveillance and clinical analytic functionalities in the context of IV catheter care are yet to be fully explored. A limitation that likely stems from the variation in uptake of EHRs to measure quality and safety on a larger health service scale (46).

Intravascular catheters are a vital device to facilitate treatment, meaning the quality of care that patients receive is an important factor affecting rates of hospital-acquired complications and infection (47). Quality standards for vascular access care exist, but at present we have limited ability to evaluate or benchmark how safe vascular access care is. The implementation of standardized IV catheter surveillance will be challenging, improving data linkage capabilities between all current systems will be costly but would increase the power to detect associations in clinical characteristics and risks. Further improved interoperability and item standardization will result in increased efficiencies which will facilitate easier, more reliable reporting and analysis of clinical and surveillance data (14). This information can be used to inform targeted catheter improvement programs, which will in turn improve patient outcomes and decrease healthcare utilization and costs.

### Implications for clinical practice and future research

We identified no clinical decision support systems working alongside or integrated with electronic platforms to support complex decision-making related to IV catheter care. This is a notable omission in the current healthcare infrastructure that future research and quality improvement efforts could seek to address, given their adoption and demonstrated benefit in other disciplines (48). In future years, clinicians and clinical informaticians will need to address fragmented ontologies and complicated exportation algorithms to make use of the currently collected data to facilitate better quality surveillance and improvement initiatives.

### Limitations and strengths

This study has limitations. First, while conducting the gray literature search, we noted platform developers provide very brief and limited information within their webpages, and not all replied to requests for more information. Our findings may therefore be incomplete. Further, while our approach was rigorous, data pertained to the healthcare system in Queensland may not be generalizable to other states, and we may not have identified all statewide datasets. Strengths of the study included consultation with experts and professionals who use these systems daily, and finally validation by clinical informaticians specializing in each of the identified platforms.

## Conclusion

Overall, we identified that current EHRs for IV catheter data collection have limited capacity to support a “learning healthcare system” where continuous improvement in indwelling IV catheter care is possible—using data to analyze and predict which treatments are more effective. While the optimal interaction design of each software is challenging, we must first address the standardization and export capacity of existing systems to optimize improvements in patient safety relative to vascular access care.

## Data availability statement

The original contributions presented in this study are included in the article/Supplementary material; further inquiries can be directed to the corresponding author.

## Ethics statement

Ethical approval was obtained from Griffith University (GU Ref: 2020/710). Written informed consent for participation was not required for this study in accordance with the national legislation and the institutional requirements.

## Author contributions

JS and CR conceived the study, designed the protocol, obtained ethical approval, contributed to data collection and analysis, wrote, and revised the final manuscript. DB conceived the study, designed the protocol, obtained ethical and governance approval, led to data collection and analysis, contributed to the writing, and revision of the final manuscript. CS, NR, GR-B, and BS contributed to protocol design, provided content expertise, contributed to the writing, and revision of the final manuscript. RW provided site-specific expertise, contributed to the refinement of the manuscript, and approved the final version for publication. All authors contributed to the article and approved the submitted version.
