# Supplementary material for: Mapping progress in intravascular catheter quality surveillance: An Australian case study of electronic medical record data linkage
Source: Front Med (Lausanne). 2022 Aug 11;9:962130. doi: 10.3389/fmed.2022.962130 (PMC9403736; doi:10.3389/fmed.2022.962130)
Supplement: Supplementary file 1 [file Table_1.DOCX]

**Supplementary Table 1.** **Research questions for systematic map**

| **Research questions for systematic map**  Object orientated Design Map  **RQ1**. Which EHRs collect IV catheter data across Queensland Health?  **RQ2**. What IV catheter data are currently collected across EHRs and how do they align with international recommendations for catheter surveillance?  **RQ3**. What clinical decision support systems (integrated clinical analytic functionalities) are currently in use to support IV catheter surveillance and care? |
| --- |
