# Supplementary material for: Mapping progress in intravascular catheter quality surveillance: An Australian case study of electronic medical record data linkage
Source: Front Med (Lausanne). 2022 Aug 11;9:962130. doi: 10.3389/fmed.2022.962130 (PMC9403736; doi:10.3389/fmed.2022.962130)
Supplement: Supplementary file 2 [file Table_2.pdf]

Vascular access minimum dataset

**Patient demographics (*n* = 5)**	**Insertion items (*n* = 16)**	**Management items (*n* = 9)**	**Complication and removal items (*n* = 15)**
1. Age	11. Indication	27. Is the device being used	36. Phlebitis
2. Weight	12. Insertion date and time	28. Site assessment	37. Infiltration & extravasation
3. Gender	13. Number of attempts	29. Lock solution	38. Primary BSI
4. Diagnostic group	14. Site of insertion	30. Dressing schedule	39. Local infection
5. Patient co-morbidities	15. Location of insertion	31. Dressing and securement	40. Dislodgement
	16. Inserter designation	32. Blood sampling	41. Thrombosis
**Device characteristics (*n* = 5)**	17. Technique used	33. Number of other VA devices	42. Occlusion
6. Device type	18. Technology used		43. Internal malposition
7. Catheter size	19. Antisepsis used	34. Complication identified	44. Fracture
8. Catheter length	20. Catheter to vein ratio	35. Use of antithrombolytics	45. Catheter associated skin injury
9. Catheter lumen	21. Tip position		46. Reason for removal
10. Catheter material	22. Tip position confirmation		47. Date time or removal
	23. Pain relief		48. Replacement insertion required
	24. Dressing and securement		49. Length of stay – hospital
	25. Insertion related adverse event		50. Patient reported pain/discomfort
	26. Can the patient identify the reason for the device?		

VA = Vascular access devices; BSI = Blood stream infection
